# Health concerns, self-control, and low-calorie food purchasing behavior

**DOI:** 10.3389/fpsyg.2026.1803573

**Published:** 2026-06-22

**Authors:** Liming Zhao, Huilin Yin, Xu Cheng, Xu Li

**Affiliations:** The College of Economics and Management, Shenyang Agricultural University, Shenyang, Liaoning, China

**Keywords:** calorie, food, health concern, purchasing behavior, self-control

## Abstract

**Objectives:**

This study examines the relationships among health concerns, self-control, and consumers’ low-calorie food purchasing behavior.

**Methods:**

A total of 320 valid electronic questionnaires were collected from respondents across various regions in China. Factor analysis was employed to calculate the composite scores of the latent variables, which were subsequently used to test the proposed hypotheses.

**Results:**

Health concerns exerted a significant positive effect on self-control (*β* = 0.14, CR = 2.08, *p* < 0.05, 95% CI [0.01, 0.29]), while self-control demonstrated a strong positive influence on low-calorie food purchasing behavior (*β* = 0.55, CR = 16.84, *p* < 0.001, 95% CI [0.43, 0.68]). However, the direct effect of health concern on purchasing behavior was not statistically significant (*β* = −0.04, CR = −1.02, *p* > 0.10, 95% CI [−0.18, 0.08]). Moreover, nutritional knowledge significantly and positively predicted self-control (*β* = 6.87, CR = 4.76, *p* < 0.001, 95% CI [3.95, 24.91]).

**Conclusion:**

This study integrates health concerns, self-control, nutritional knowledge, and low-calorie food purchasing behavior into a unified theoretical framework, thereby deepening the understanding of the mechanisms through which health concerns influence consumers’ low-calorie food purchasing behavior.

## 1 Introduction

Obesity is a major public health challenge with profound implications for human health worldwide. According to the World Obesity Atlas 2024 (World Obesity Federation, 2024), global estimates based on body mass index (BMI) indicate that approximately 2.2 billion adults were classified as overweight (25 ≤ BMI < 30 kg/m^2^) or obese (BMI ≥ 30 kg/m^2^) in 2020, accounting for approximately 40% of the global adult population. The report further projects that by 2035, more than half of all adults worldwide will be overweight or obese.

Being overweight or obese not only increases the risk of numerous health problems but also imposes substantial economic and healthcare burdens on societies. Previous studies have shown that excess body weight is strongly associated with chronic diseases such as diabetes, hypertension, and cardiovascular disease (Harris-Lagoudakis, 2022; Seligman et al., 2007, 2010). These conditions contribute to rising healthcare expenditures and place increasing pressure on healthcare systems across countries and regions (Seligman et al., 2007; Zhu et al., 2016).

The causes of overweight and obesity are multifaceted. In addition to genetic factors, they are closely associated with work patterns and lifestyle habits, particularly the excessive consumption of foods high in fat, sugar, and calories. To reduce excessive calorie intake, some countries and regions have implemented regulations requiring chain restaurants to display calorie information on menus (Bollinger et al., 2011; Kim and Nicolau, 2021).

However, the effects of calorie disclosure on consumers’ low-calorie food purchasing behavior remain inconsistent. Some studies suggest that calorie disclosure reduces the average calorie content of foods purchased by consumers (Zlatevska et al., 2018), whereas others indicate that calorie disclosure may increase the average calorie content of purchased foods (Lewis et al., 2016). Additional studies report no significant effect of calorie disclosure on consumers’ purchasing behavior (Bollinger et al., 2011; Breck et al., 2017). These inconsistent findings present significant challenges for public policymakers. A key issue is how to identify and target consumers who are most responsive to health-related interventions and how to communicate the risks associated with excessive calorie intake to mitigate public health crises.

To address this issue, the present study focuses on consumers’ health concerns and related knowledge. Specifically, it proposes that consumers with greater health concerns are more likely to actively monitor their dietary behaviors. Moreover, consumers require sufficient knowledge regarding the relationship between calorie intake and health outcomes to make healthier dietary choices. The study further proposes that consumers with stronger health concerns are more likely to purchase low-calorie foods and that this relationship is mediated by self-control. Furthermore, consumers’ level of health-related knowledge is expected to strengthen this relationship.

This study’s findings contribute to the advancement of related theories. Previous literature has suggested that strong health motivation is an important predictor of healthy or low-calorie food choices (Breck et al., 2017; Bayliss, 2025; Keller and Lehmann, 2008). Building on this line of research, the present study introduces self-control to further extend the theoretical framework. Self-control refers to an individual’s ability to resist immediate impulses in response to tempting stimuli (Hassan et al., 2010). Accordingly, this study develops an integrated theoretical framework linking motivation, ability, self-control, and healthy food choice behavior, thereby laying a solid foundation for future research in this area.

### 1.1 Literature review

This study examines the relationship between health concerns and low-calorie food purchasing behavior. To provide a more comprehensive understanding of this issue, the study also considers the mediating role of self-control and the influence of nutritional knowledge. Table 1 summarizes the main findings of prior studies related to these variables.

**Table 1:** Summary of related literature.

	Variables				Main conclusions
	Health concerns	Nutritional knowledge	Self-control	Low-calorie food purchase behavior/Healthy eating	
Souza-Monteiro et al. (2022)	•			•	Consumers with health issues are more likely to make healthier food choices.
Berry et al. (2019)	•			•	Calorie labels are more effective for consumers with high health motivation, encouraging them to choose low-calorie foods.
Drywień et al. (2021)	•			•	A positive correlation does not always exist between health issues and low-calorie food purchase behavior/healthy eating.
Mancone et al. (2024)		•		•	With improved food knowledge, the healthy eating habits of teenagers have significantly improved.
Leschewski et al. (2018)		•		•	Families who have received nutrition education are no more likely to purchase low-calorie foods than are those who have not.
Bower et al. (2003)	•	•		•	Health concerns and nutritional knowledge appear to have a positive interactive effect on purchase intent.
Zhang et al. (2010)			•	•	Consumers with stronger self-control tend to view temptation as a major obstacle to achieving their goals, making them more likely to resist temptation and choose low-calorie foods.
Sleboda et al. (2025)			•	•	Self-control is positively correlated with healthy food purchase behavior.
Current research	•	•	•	•	Health concerns enhance consumers’ self-control, thereby positively influencing their low-calorie food purchasing behavior.
					Consumers’ nutritional knowledge moderates the relationship between self-control and low-calorie food purchasing behavior.

According to the dominant perspective in the health literature, consumers with higher levels of health concern, health awareness, or health motivation are more likely to purchase low-calorie foods. For instance, Ailawadi et al. (2018) analyzed consumers’ shopping behavior and found that club stores significantly increase consumers’ food purchases, a phenomenon they termed “the club store effect.” However, further analysis showed that households with higher incomes or stronger health concerns were less susceptible to this effect. Souza-Monteiro et al. (2022) analyzed the impact of food calorie counters in online shopping baskets and found that these tools exerted a stronger positive influence on consumers with health-related problems. Similarly, Berry et al. (2019) found that calorie labels effectively reduced the number of calories ordered by health-conscious consumers. In contrast, Drywień et al. (2021) and Samdal et al. (2022) reported either no relationship or a negative relationship between health concerns and low-calorie food purchasing behavior.

Regarding health knowledge, Samdal et al. (2022) and Mancone et al. (2024) believe that as consumers’ understanding of food and health knowledge increases, their dietary habits will also improve. However, Leschewski et al. (2018) reported contrasting findings, showing families with greater health education did not consume fewer calories when dining out than families with less health education. Bower et al. (2003) simultaneously examined the effects of health concerns and nutritional knowledge on healthy food purchasing behavior and found that consumers with higher levels of health concern or nutritional knowledge were more likely to choose healthy foods. Moreover, the study identified a significant positive interaction effect between health concerns and nutritional knowledge in predicting healthy foods. Overall, the existing literature suggests that the relationship between nutritional knowledge and healthy eating or low-calorie food purchasing behavior is complex.

Drichoutis et al. (2006) suggested that low-calorie food selection or indulgent food consumption are closely associated with self-control, although they did not fully explore the underlying theoretical mechanisms. Zhang et al. (2010) found that consumers often resolve conflicts between long-term goals and short-term temptations by interpreting temptations. Specifically, when facing self-control conflicts, consumers may intentionally perceive temptations as more harmful to their long-term goals, thereby helping themselves resist immediate gratification. Moreover, Cobb-Clark et al. (2023) and Sleboda et al. (2025) demonstrated a positive relationship between self-control and healthy eating behaviors.

The existing literature provides an essential theoretical and empirical foundation for understanding healthy eating and low-calorie food purchasing behavior. Some studies suggest that health concerns are positively associated with healthy eating and low-calorie food purchases (Berry et al., 2019; Souza-Monteiro et al., 2022), whereas others argue that this relationship is inconsistent or context-dependent (Drywień et al., 2021). Similarly, although some studies indicate that consumers with greater health knowledge are more likely to purchase low-calorie foods (Mancone et al., 2024), other studies have reported conflicting findings (Leschewski et al., 2018). For instance, dining out— particularly in family settings—is often motivated by enjoyment and social bonding, creating more opportunities for indulgence, making self-control an important factor. Therefore, self-control may play a critical role in explaining the relationship between health concerns and low-calorie food purchasing behavior.

However, the existing literature on health concerns and low-calorie food purchasing behavior has largely overlooked the role of self-control. Similarly, studies focusing on self-control or nutritional knowledge have generally failed to account for the influence of health concerns. To address these gaps, this study integrates health concerns, self-control, nutritional knowledge, and low-calorie food purchasing behavior into a unified theoretical framework. This integrated approach contributes to the advancement of the theoretical literature and provides both theoretical and empirical evidence regarding the conditions under which health concerns are likely to promote consumers’ low-calorie food purchasing behavior.

### 1.2 Research framework and hypothesis development

Research on food, nutrition, and health has received considerable attention from scholars in medicine, public health, marketing, and agricultural economics (Costlow et al., 2026; Huang and Lee, 2023; Pomeranz, 2025). These studies can be broadly classified into two main categories: (1) how to ensure that low-income populations have access to sufficient food to meet their basic nutritional and health needs and (2) how to address public health issues related to the excessive intake of calories and nutrients. In other words, both undernutrition and overnutrition can lead to public health problems. This study is aligned with the second area of focus, specifically investigating whether and how consumers’ health concerns influence their low-calorie food purchasing behavior.

**Figure 1:**
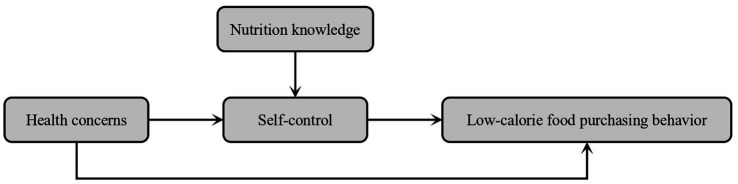
Research framework.

The extant literature on consumer behavior suggests that the decision to purchase low-calorie foods is typically driven by a strong motivation to maintain health and a good level of nutritional knowledge (Choi and Samper, 2019; Leschewski et al., 2018; Zhang et al., 2010). Specifically, health motivation is closely associated with self-control (Prinsen et al., 2018; Zhang et al., 2010) and health concern (Bower et al., 2003; Souza-Monteiro et al., 2022). Building on this theoretical foundation, the present study develops the research framework shown in Figure 1, which includes four key variables: health concern, self-control, nutritional knowledge, and low-calorie food purchasing behavior. Within this framework, the study explores three key topics, as described below.

#### 1.2.1 Health concerns and low-calorie food purchasing behavior

Individuals are becoming increasingly concerned about their health, particularly those who are currently experiencing health problems or have previously encountered them (de Ridder et al., 2017). These individuals are also more aware of the benefits associated with healthy dietary habits. Souza-Monteiro et al. (2022) examined the relationship between online shopping behavior and low-calorie food choices and found a significant positive relationship between health concerns and the likelihood of purchasing low-calorie foods. Similarly, Ailawadi et al. (2018) demonstrated that consumers with stronger health concerns exhibit greater preferences for foods with lower calorie content. Health concern refers to the degree to which individuals are concerned about their health and value maintaining good health (Gebhardt et al., 2001). Low-calorie food purchasing behavior refers to the likelihood that individuals will purchase low-calorie foods (Wang et al., 2025; Zhao et al., 2024). Based on these findings, the following hypothesis is proposed:

*H_1_*: Individuals with higher health concerns are more likely to purchase low-calorie foods.

#### 1.2.2 The mediating role of self-control

Although individuals are often tempted by palatable foods, calorie intake must be regulated to avoid negative consequences such as weight gain. In this context, effective self-control is necessary to resist the temptation of high-calorie foods. Achieving this requires not only awareness but also strong motivation and willpower (Fishbein and Cappella, 2006; Keller and Lehmann, 2008). Self-control refers to individuals’ overall ability to regulate their food consumption effectively (Tangney et al., 2004). The current research proposes that individuals who are highly health-conscious or who have experienced significant health problems are more motivated to regulate their diets and, consequently, more likely to purchase low-calorie foods. This is because such individuals are more aware of the severe health risks associated with excessive calorie intake.

The underlying psychological mechanism can be explained by the self-control theory proposed by Zhang et al. (2010), which suggests that self-control operates through a process of “counteractive construal.” When individuals face a conflict between immediate desires (i.e., the temptation of delicious, high-calorie food) and long-term goals (i.e., maintaining a healthy weight), they tend to exaggerate the negative consequences of in short-term temptations for their long-term objectives. Consequently, they become motivated to resist temptation and remain committed to their long-term health goals.

Building on this theoretical perspective, the present study proposes that more health-conscious individuals have greater self-control, which enables them to resist tempting foods more effectively. Self-control is therefore expected to mediate the relationship between health concerns and low-calorie food purchasing behavior. In other words, self-control functions as a critical psychological mechanism through which health concerns influence food choices. Accordingly, the following hypothesis is proposed:

*H_2_*: Self-control serves as a mediator in the relationship between health awareness and low-calorie food purchasing behavior. Specifically, (a) individuals with greater health consciousness are more likely to exhibit higher levels of self-control and (b) stronger self-control is associated with an increased likelihood of purchasing low-calorie foods.

#### 1.2.3 The effect of nutritional knowledge

Nutritional knowledge refers to individuals’ understanding of the relationship between food, calorie intake, and health outcomes or disease. Effective dietary control requires not only strong motivation and willpower (Berman and Lavizzo-Mourey, 2008; Howlett et al., 2009), but also the ability to accurately understand the relationship between diet and health outcomes (Souza-Monteiro et al., 2022). In other words, individuals must possess sufficient nutritional knowledge regarding how food consumption, particularly calorie intake, affects health and disease. Without such knowledge, motivation to regulate dietary behavior is unlikely to be translated into informed, healthy purchasing behavior.

In practice, even when consumers are motivated to make healthier choices, they must first obtain and understand relevant nutrition information before integrating it into their purchasing decisions. This highlights the importance of nutritional knowledge as a critical factor that not only enables informed decision-making but also strengthens individuals’ ability to exercise self-control when confronted with tempting food options. Accordingly, the following hypothesis is proposed:

*H_3_*: Nutritional knowledge has a positive impact on self-control.

## 2 Materials and methods

### 2.1 Data

A power analysis was conducted using GPower 3.1.9.7 to determine the required sample size for the study. Based on previous research examining relationships between media use and psychological variables, a small effect size (correlation *|ρ|* = 0.20) was specified. Using a two-tailed test, an alpha level of 0.05, and a desired power (1–*β*) of 0.95, the analysis indicated that a minimum sample size of 319 participants was required.

The survey was conducted in March 2026 using Wenjuanxing, a Chinese online survey tool. Online surveys are widely used in academic research and practical applications because they provide efficiency, accessibility, and scalability in data collection (Infanti et al., 2024; Spantios et al., 2025). Prior to data collection, the study protocol was reviewed and approved by the Ethics Committee of the authors’ affiliated institution (Approval No. SYAU-2026-033), ensuring compliance with relevant ethical and legal standards.

The survey link was distributed through social media platforms. All participants were adults residing in China. Before completing the questionnaire, participants were presented with an informed consent form explaining the study purpose, the voluntary nature of participation, and the confidentiality of responses. Only participants who provided informed consent proceeded to the questionnaire. As an incentive, participants were entered into a random drawing for a cash reward of 5 yuan after completing the survey. Such random incentives are commonly used in survey research. A total of 383 responses were collected. However, 63 questionnaires were excluded because they were completed in less than 90 s, whereas the research team estimated that at least 90 s were required to complete the questionnaire adequately. Consequently, 320 valid questionnaires were retained for hypothesis testing.

The questionnaire consisted of five sections covering the core variables in the research framework: low-calorie food purchasing behavior, health concerns, self-control, nutritional knowledge, and demographic characteristics. Table 2 presents a detailed description of the survey items and variables. Regarding demographic characteristics, 46.2% of the participants were male and 53.8% were female. Most The participants were younger than 40 years old, accounting for 81% of the sample. In terms of annual household income, 15% reported incomes below 50,000 yuan, 24% reported incomes between 60,000 and 100,000 yuan, 25% reported between 110,000 and 150,000 yuan, and 36% reported incomes 160,000 yuan or above.

**Table 2:** Reliability and validity.

Latent variables	Observed variables	SFL	CR	AVE	HTMT			
					(PB)	(SC)	(HC)	(NK)
Low-calorie food purchasing behavior (PB)	In my daily life, I often purchase low-calorie foods.	0.84	0.87	0.69	1			
	Within my acceptable price range, I am willing to pay a higher price for low-calorie foods.	0.74						
	In the future, I will continue to purchase low-calorie foods.	0.91						
Self-control (SC)	When eating meals, I often reduce my food intake to control my weight.	0.90	0.92	0.80	0.56	1		
	When eating meals, I often restrain myself to prevent weight gain.	0.92						
	I often limit my food intake to control my weight.	0.87						
Health concerns (HC)	I believe my weight has seriously exceeded the normal range.	0.64	0.88	0.64	0.06	0.15	1	
	I believe my blood sugar level has seriously exceeded the normal range.	0.84						
	I believe my blood pressure has seriously exceeded the normal range.	0.83						
	I believe my blood lipid levels have seriously exceeded the normal range.	0.87						
Nutritional knowledge (NK)	I believe that excessive calorie intake leads to obesity.	0.79	0.85	0.66	0.21	0.15	0.09	1
	I believe that excessive calorie intake may cause heart disease.	0.80						
	I believe that excessive calorie intake is likely to cause the “three highs.”	0.85						
Model Fit Indices	CFI > 0.95; RMSEA ≤ 0.08; SRMR < 0.08; χ^2^/df = 2.75							

Given that AMOS does not directly provide Heterotrait-Monotrait (HTMT) statistics, correlation coefficients between latent variables were used as an approximation. All values were below 0.85, suggesting satisfactory discriminant validity. SFL, Standardized Factor Loadings; CR, Composite Reliability, and AVE, Average Variance Extracted.

### 2.2 Variable settings

This study examines the influence of health concerns on consumers’ low-calorie food purchasing behavior, with a focus on the mediating role of self-control and the effect of nutritional knowledge. To measure these variables, the study adapted established scales from previous research. The measurement scale was adapted from well-validated and widely recognized scales published in leading academic journals; therefore, a pilot study was not deemed necessary (Alexander and Tepper, 1995; Bower et al., 2003; Kähkönen et al., 1996; Stice, 1998; Zhao et al., 2024).

The dependent variable, low-calorie food purchasing behavior, was measured using a 5-point Likert scale (1 = strongly disagree, 5 = strongly agree) based on Zhao et al.’s (2024) approach. Participants indicated their agreement with statements such as: “I often purchase low-calorie foods”; “Within my acceptable price range, I am willing to pay a higher price for low-calorie foods”; and “In the future, I will continue to purchase low-calorie foods (including beverages).” The key explanatory variable, health concerns, was measured using four items adapted from Kähkönen et al. (1996). These items measured participants’ concerns regarding weight, blood sugar, blood pressure, and general well-being using a 5-point Likert scale. Examples include: “I believe my weight has seriously exceeded the normal range” and “I believe my blood pressure has seriously exceeded the normal range.”

The mediating variable, self-control, was measured using a three-item scale adapted from Stice (1998). All items were assessed on a 5-point Likert scale, including statements such as: “When eating meals, I often reduce my food intake to control my weight.” Nutritional knowledge was measured through five items adapted from Alexander and Tepper (1995) and Bower et al. (2003) that assessed participants’ beliefs about the health consequences of excessive calorie intake. These items included “I believe that excessive calorie intake leads to obesity” and “I believe that excessive calorie intake may cause heart disease.”

Finally, demographic characteristics were included as control variables to account for potential confounding factors, as prior studies have shown that such demographic factors may influence consumers’ food-related purchasing behaviors (Ameri et al., 2023; Gao et al., 2025; Zlatevska et al., 2018). They included sex, age, occupation, household income, and educational background. Sex was categorized as male or female, age was divided into 10 groups ranging from under 20 years to 61 years and above, educational attainment ranged from primary school to graduate school, and household income was divided into five categories based on annual income in RMB.

### 2.3 Reliability and validity tests

#### 2.3.1 Reliability test

Reliability analysis was conducted to assess the internal consistency of the measurement instruments and ensure the reliability of the collected data. In this study, Cronbach’s *α* coefficient was used to evaluate the reliability of the questionnaire. Higher Cronbach’s α values indicate greater internal consistency and reliability of the measurement scale. Generally, an *α* value below 0.60 indicates low reliability and suggests that the questionnaire requires improvement; values between 0.60 and 0.70 indicate acceptable reliability; and values above 0.70 indicate high reliability and strong internal consistency. The Cronbach’s α coefficients for low-calorie food purchasing behavior, health concerns, self-control, and nutritional knowledge were 0.85, 0.85, 0.92, and 0.85, respectively. Because all values exceeded 0.80, the questionnaire demonstrated high reliability and satisfactory internal consistency.

#### 2.3.2 Validity test

Validity analysis was conducted to examine whether the measurement scales accurately captured the constructs and characteristics of the variables under investigation, thereby ensuring the accuracy and effectiveness of the measurements. This study used SPSS 26.0 to conduct the validity analysis. Specifically, factor analysis was performed using principal component analysis for factor extraction, followed by varimax rotation. The Kaiser–Meyer–Olkin test results of low-calorie food purchasing behavior, health concerns, self-control, and nutritional knowledge were 0.70, 0.81, 0.75, and 0.72, respectively. All results were statistically significant at the 0.001 level, indicating that the scales possessed adequate validity and were appropriate for further analysis.

## 3 Results

Confirmatory factor analysis was conducted using AMOS 26.0 to validate the measurement model. The results indicated a satisfactory model fit: CFI = 0.95, TLI = 0.92, RMSEA = 0.06, SRMR = 0.05, and χ^2^/df = 2.75. Convergent validity was supported by standardized factor loadings above 0.6, composite reliability (CR) values greater than 0.70, and average variance extracted values exceeding 0.50 for all constructs. Discriminant validity was also established, all Heterotrait-Monotrait Ratio values were below 0.85, confirming adequate distinctiveness among the constructs. Collectively, these results satisfy contemporary psychometric standards and provide strong support for the reliability and validity of the measurement model.

This study proposes three hypotheses to examine the relationships among health concern, self-control, nutritional knowledge, and low-calorie food purchasing behavior. Specifically, it hypothesizes that consumers with greater health concerns are more likely to engage in low-calorie food purchasing behavior (H_1_). Moreover, the study proposes that self-control mediates the relationship between health concern and low-calorie food purchasing behavior (H_2a_ and H_2b_), and that nutritional knowledge exerts a significant positive effect on consumers’ self-control (H_3_). Path analysis was conducted using IBM SPSS AMOS to test these hypotheses.

**Figure 2:**
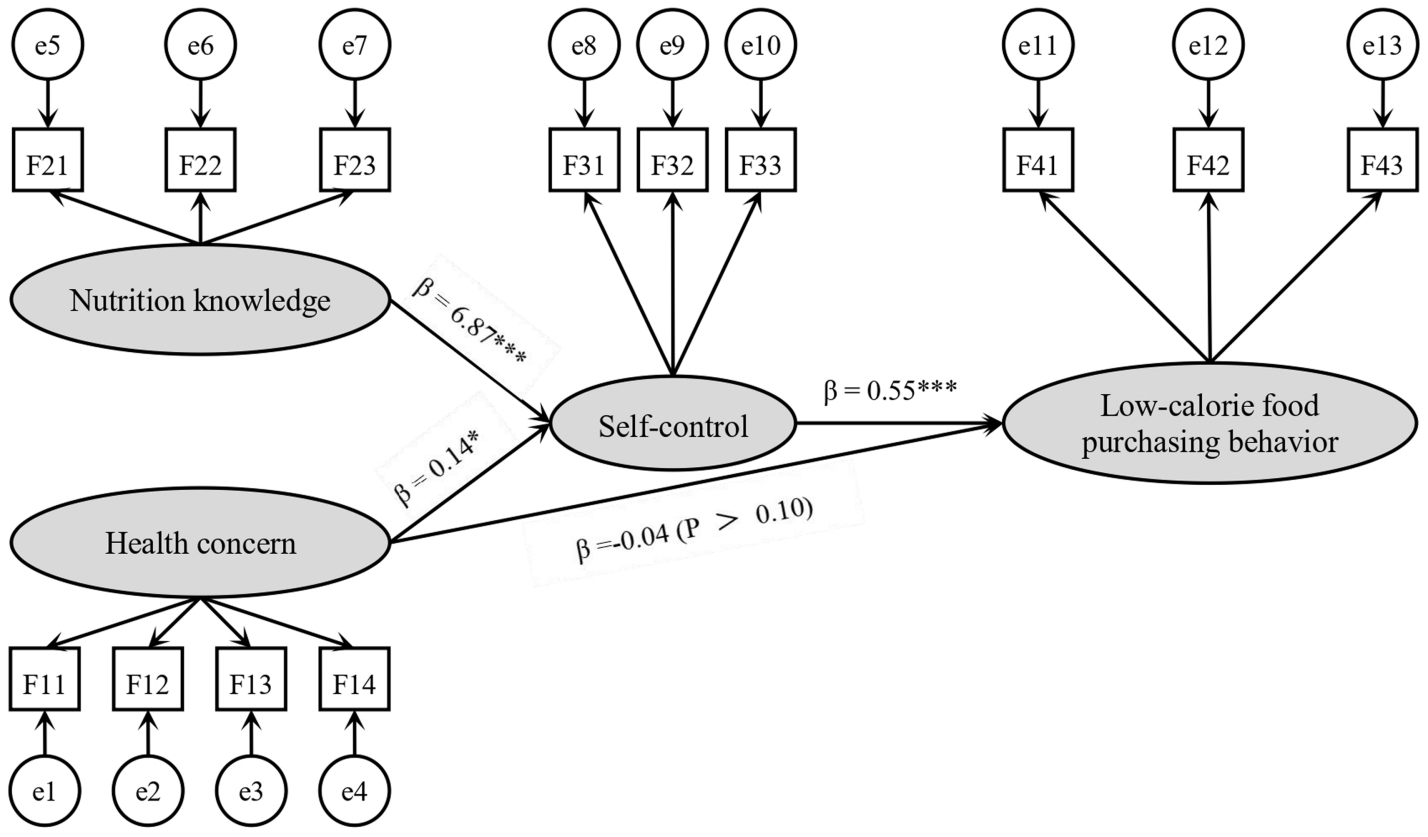
Path analysis results. *p ≤ 0.05, **p ≤ 0.01, ***p ≤ 0.001.

The path analysis results revealed several key findings regarding the hypothesized relationships, as illustrated in Figure 2. First, health concerns exerted a significant positive effect on self-control (*β* = 0.14, CR = 2.08, *p* < 0.05, 95% CI [0.01, 0.29]), while self-control demonstrated a strong positive influence on low-calorie food purchasing behavior (*β* = 0.55, CR = 16.84, *p* < 0.001, 95% CI [0.43, 0.68]). In contrast, the direct effect of health consciousness on purchasing behavior was not statistically significant (*β* = −0.04, CR = −1.02, *p* > 0.10, 95% CI [−0.18, 0.08]), suggesting that self-control fully mediates the relationship between health consciousness and low-calorie food purchasing behavior. Accordingly, Hypotheses H_2a_ and H_2b_ are supported, whereas Hypothesis H1 is not supported. Second, consistent with the proposed hypothesis, nutritional knowledge significantly and positively predicted self-control (*β* = 6.87, CR = 4.76, *p* < 0.001, 95% CI [3.95, 24.91]), thereby supporting Hypothesis H3. Collectively, these findings highlight the pivotal role of self-control in shaping low-calorie food purchasing behavior and underscore the positive influence of nutritional knowledge on enhancing self-control.

To systematically examine the mediating mechanism, this study employed the bias-corrected bootstrap procedure with 5,000 resamples and 95% CIs in AMOS 26 to test the mediating effect of self-control, following the methodological guidelines proposed by Preacher and Hayes (2008).

Based on the original structural equation modeling results, the study reported the total, direct, and indirect effects. The unstandardized total effect of health concern on low-calorie food purchasing behavior was 0.04. After introducing self-control as the mediating variable, the direct effect of health concern on purchasing behavior became −0.04 and was not statistically significant (*p* = 0.31 > 0.10). In contrast, the unstandardized indirect effect of health concern on purchasing behavior through self-control was 0.08, and the corresponding 95% bias-corrected bootstrap CI did not include zero, thereby confirming a significant mediating effect.

Regarding the specific path coefficients, health concern positively and significantly influenced self-control (estimate = 0.14, *p* = 0.04), while self-control exerted a strong positive effect on consumers’ low-calorie food purchasing behavior (estimate = 0.55, *p* < 0.001). Taken together, these findings confirm the presence of full mediation. Specifically, health concern does not directly influence consumers’ low-calorie food purchase decisions, its effect is fully transmitted through the mediating role of self-control.

## 4 Discussion

In recent years, growing public awareness of obesity, chronic diseases, and other diet-related health issues has increased consumers’ interest in low-calorie and healthier food options. Despite this trend, the prevalence of overweight and obesity continues to rise (World Obesity Federation, 2024). To address these serious public health concerns, many countries and regions have required restaurants and food manufacturers to display calorie information on food packaging and menus. Interestingly, some studies have found that consumers make healthier or low-calorie dietary choices based on calorie labeling, whereas others have reported the opposite findings. This inconsistency poses a significant challenge to public health management. A key question, therefore, is how to effectively identify and target consumers who are most responsive to health-related interventions and how to communicate the risks of excessive calorie intake to mitigate a public health crisis.

This study investigates consumers’ low-calorie food purchasing behavior, with a particular focus on the role of health concerns. The analysis examines the relationship between health concerns and low-calorie food purchasing behavior by incorporating the mediating role of self-control and the effect of nutritional knowledge. The results indicate that higher levels of health concern enhance consumers’ self-control, which subsequently increases their likelihood of purchasing low-calorie foods. Furthermore, consumers with greater nutritional knowledge demonstrate stronger self-control.

### 4.1 Theoretical contributions

This study advances the theoretical development of the literature on health concerns and low-calorie food purchasing behavior. Previous studies have overlooked the importance of self-control, while studies examining the relationship between self-control and low-calorie food purchasing behavior have largely ignored the role of health concerns. By integrating health concerns, self-control, health knowledge, and low-calorie food purchasing behavior into a single research framework, this study contributes to the theoretical advancement of the related literature. The findings reveal that self-control mediates the relationship between health concerns and low-calorie food purchasing behavior, indicating that health concerns alone are insufficient to drive healthier purchasing decisions and that effective self-control is also required. Moreover, nutritional knowledge positively moderates the influence of self-control on low-calorie food purchasing behavior, suggesting that it strengthens the effectiveness of self-control in shaping healthy consumption behavior.

The proposed framework also provides a plausible explanation for the inconsistent findings reported in previous studies. Some studies have shown that health concerns positively influence consumers’ purchases of low-calorie foods and adoption of healthier diets (Ailawadi et al., 2018; Berry et al., 2019; Souza-Monteiro et al., 2022). In contrast, Drywień et al. (2021) and Samdal et al. (2022) found either no relationship or a negative relationship between health concerns and low-calorie food purchasing behavior.

Regarding nutritional knowledge, some studies suggest that greater knowledge about food and health improves dietary habits (Samdal et al., 2022; Mancone et al., 2024), whereas others report that health education does not reduce calorie intake when dining out (Leschewski et al., 2018). The framework proposed in this study helps explain these contradictory findings by demonstrating that health concerns influence low-calorie food purchasing behavior through self-control. In other words, without sufficient self-control, health concerns are unlikely to translate into healthier purchasing behavior. Moreover, nutritional knowledge represents another important influencing factor; without adequate nutritional knowledge, the ability of self-control to promote low-calorie food purchasing behavior may be weakened.

### 4.2 Practical implications

This study’s findings carry important implications for public health authorities, policymakers, and marketers seeking to promote healthier eating behaviors. First, regarding public health promotion, the results indicate that health awareness positively influences consumers’ low-calorie food purchasing behavior through self-control, suggesting that health awareness functions as a motivational factor that encourages behavioral change. Accordingly, public health agencies should design interventions that strategically target individuals with heightened health awareness or health-related concerns. For example, promotional materials emphasizing the benefits of calorie control and the risks associated with excessive calorie intake could be placed in hospitals, pharmacies, and other healthcare settings where health-conscious individuals are more likely to encounter them. Such context-specific communication may effectively encourage consumers to translate their concerns into actual behavioral changes. The findings also confirm that nutritional knowledge strengthens self-control. Therefore, educational campaigns aimed at improving consumers’ understanding of calorie content, nutrition labeling, and the health consequences of dietary choices are essential. Public health authorities should develop clear, accessible, and evidence-based informational materials to enhance public nutrition literacy. By fostering greater awareness of the relationship between calorie intake and health, these initiatives can empower consumers to apply self-control more effectively in food-related decisions. These recommendations are consistent with the arguments advanced by Bos et al. (2018), Cadario and Chandon (2020), and Berry et al. (2019) regarding the importance of targeted health interventions that account for consumer heterogeneity.

Second, the findings provide several crucial implications for the food industry and marketing practitioners. Since consumers’ health concerns serve as an essential motivational driver of low-calorie food purchases, food companies should develop marketing strategies that emphasize health benefits and risk reduction. Messages aligned with consumers’ health values—such as obesity prevention, energy balance, and long-term wellness—may strengthen consumers’ motivation to choose healthier products. For instance, companies could highlight scientific evidence supporting nutritional value, use credible health claims, and adopt transparent labeling practices to enhance consumer trust and reinforce health-related motivations. Given the mediating role of self-control, marketers should also implement strategies that facilitate consumers’ self-regulatory processes at the point of decision-making. This can be achieved by simplifying the choice environment through clear calorie labeling, portion-controlled packaging, and the strategic placement of low-calorie products in prominent retail store locations. Digital interventions, such as personalized reminders and goal-tracking features integrated into mobile applications, may also help consumers maintain self-control in real purchasing contexts. Finally, the role of nutritional knowledge further highlights the importance of consumer education and information accessibility. Food companies and public health agencies can collaborate to develop educational campaigns that improve consumers’ understanding of nutrition information and dietary balance. Marketing initiatives that incorporate informative and engaging content—such as recipe ideas, nutritional comparisons, or QR codes linked to verified health information—can further empower consumers to make more informed dietary decisions.

### 4.3 Limitations and directions for future research

Despite its theoretical and practical contributions, this study has several limitations that suggest important directions for future research. First, the study relies on cross-sectional self-report data, which limits inferences regarding the temporal ordering and causal relationships among health concerns, self-control, and purchasing behavior. Future studies could adopt longitudinal or experimental designs to establish causality and examine how changes in health concerns over time influence self-control and purchasing patterns. Moreover, incorporating objective behavioral measures, such as actual purchase records or incentivized experimental choice tasks, would improve the robustness and ecological validity of the findings.

Second, although the proposed model integrates motivation, knowledge, and self-control, other psychological constructs, such as habit strength, perceived behavioral control, and food-related identity, may also interact with self-control in shaping purchasing behavior and healthy consumption. Future studies should examine these additional mediating and moderating factors to develop a more comprehensive model of health-oriented consumption.

Third, the study sample may reflect specific cultural and socioeconomic characteristics that limit the generalizability of the findings. Cultural norms related to diet, body image, and health awareness may influence both the level of health concern and the expression of self-control. Comparative studies across different cultural contexts could provide further insight into how societal factors shape the relationships identified in this study.

In conclusion, this study provides both theoretical and practical insights into the mechanisms underlying consumers’ low-calorie food purchasing behavior. By identifying health concerns as a motivational antecedent, self-control as a mediating mechanism, and nutritional knowledge as an important influencing factor, the study offers an integrative framework linking motivation (health), ability (self-control), cognition (knowledge), and behavior. This framework advances existing theories of consumer health decision-making and provides actionable guidance for public health initiatives and marketing strategies aimed at promoting healthier dietary choices.

## Data Availability

The datasets presented in this article are not readily available because in accordance with the ethical agreement signed with research participants, we committed not to disclose or share individual-level data with any third party to protect participant confidentiality. Therefore, public deposition of the dataset is not possible. Requests to access the datasets should be directed to LZ: 2015500019@syau.edu.cn.
